# Multistakeholder perspectives on the mistreatment of indigenous women during childbirth in Colombia: drivers and points for intervention

**DOI:** 10.1186/s12884-022-04495-4

**Published:** 2022-03-11

**Authors:** Emily Gaffney Gleason, Jennifer Marcela López Ríos, Diana Patricia Molina Berrío, Cristina Mejía Merino

**Affiliations:** 1 Fulbright Commission of Colombia, Calle 37 #15-73, Medellin, Antioquia Colombia; 2 University of Pennsylvania Perelman School of Medicine, 3400 Civic Center Blvd, Philadelphia, PA 19104 USA; 3 Universidad de Antioquia, Facultad Nacional de Salud Pública, Calle 62 #52-59, Medellin, Antioquia Colombia

**Keywords:** Maternal health, Disrespect and abuse, Mistreatment, Childbirth, Colombia, Indigenous communities, Internal displacement

## Abstract

**Background:**

Abusive and disrespectful treatment of women during childbirth is a critical global issue that threatens women’s sexual rights and reproductive rights and access to quality maternal care. This phenomenon has been documented in Colombia. However, little emphasis has been placed on identifying the drivers of and potential interventions against disrespect and abuse against particularly vulnerable populations in the country, including internally displaced indigenous women.

**Methods:**

This report is a sub-analysis of a larger project. Semi-structured interviews were conducted with indigenous (Embera) women with childbirth experience (*n* = 10), maternal healthcare workers (*n* = 6), and community stakeholders (*n* = 5) in Medellín, Colombia. Qualitative analysis techniques, consisting of inductive and deductive approaches, were used to identify and characterize the drivers of disrespect and abuse against indigenous women during childbirth and points for intervention. Existing frameworks were adapted to thematically organize drivers and potential solutions into four interrelated subsystems: individual and community factors, clinician factors, facility factors, and national health system factors.

**Results:**

Participants highlighted disrespect and abuse as stemming from (within the individual and community level) its normalization, lack of autonomy and empowerment among indigenous women, lacking antenatal care, (within the clinician level) prejudice, linguistic or cultural barriers to communication, lack of understanding of indigenous culture, medical culture and training, burnout and demoralization, (within the facility level) inadequate infrastructure, space, and human resources, and (within the national systems level) lack of clear policies and the devaluing of respectful maternity care. They called for interventions specific to these drivers, grounded in dignity and respect for indigenous culture.

**Conclusion:**

This paper expands upon the growing literature on global mistreatment during childbirth by highlighting drivers of mistreatment and identifying points for intervention in a previously unstudied population. Our data show that indigenous women are especially vulnerable to mistreatment due to cultural and linguistic barriers and prejudice. Broad and meaningful action is urgently needed to realize these women’s rights to respectful maternity care. Interventions must be multifaceted and locally specific, taking into account the needs and wants of the women they serve.

**Supplementary Information:**

The online version contains supplementary material available at 10.1186/s12884-022-04495-4.

## Resumen

**Contexto:**

El trato abusivo recibido por mujeres durante el parto es un problema mundial que amenaza los derechos de las mujeres y el acceso a una atención materna de calidad. Este fenómeno ha sido documentado en Colombia, pero poco énfasis se ha hecho en poblaciones vulnerables, como las mujeres indígenas desplazadas.

**Metodología:**

Se presentan resultados derivados de un proyecto macro. Se realizaron entrevistas con mujeres indígenas Emberas que tuvieron experiencia de parto en Medellín (*n* = 10), profesionales de salud (*n* = 6) y líderes comunitarios (*n* = 5). Se utilizaron técnicas de análisis cualitativo de enfoques inductivos y deductivos, para identificar aspectos que generan el maltrato contra las mujeres indígenas durante el parto y posibles intervenciones para evitar su ocurrencia. Los modelos teóricos existentes se adaptaron para organizar temáticamente los generadores del maltrato y soluciones en cuatro subsistemas: factores individuales y comunitarios, factores clínicos, factores relacionados a los hospitales y factores asociados al sistema nacional.

**Resultados:**

Los participantes destacaron como factores a nivel individual y comunitario la normalización de tratos irrespetuosos y abusivos de parte de los profesionales de salud con las mujeres indígenas, así como su falta de autonomía y empoderamiento. A nivel clínico, se identificó la falta de atención prenatal, barreras para la comunicación, falta de comprensión de la cultura indígena, la cultura y formación médica, la desmoralización de los médicos. Sobre los servicios de salud, se encontraron infraestructuras, espacios y recursos humanos inadecuados. En el nivel del sistema, se identifican la falta de políticas y poca valoración de la atención respetuosa. Los participantes señalaron la importancia de realizar intervenciones sobre estos generadores, basados en la dignidad y el respeto por la cultura indígena.

**Conclusiones:**

Este artículo amplía la creciente literatura global sobre el maltrato dirigido a las maternas durante el parto y muestra que las mujeres indígenas son especialmente vulnerables debido a las barreras y prejuicios culturales y lingüísticos. Se necesitan urgentemente acciones amplias para hacer realidad los derechos de las mujeres frente a la atención materna respetuosa. Las intervenciones deben ser multifacéticas y específicas a nivel local, teniendo en cuenta las necesidades y deseos de las mujeres a las que sirven.

## Background

As clinical indicators of maternal health improve worldwide, increased emphasis has been placed upon quality of care to improve maternal health outcomes, reduce maternal mortality, and ensure the fulfilment of women’s sexual rights and reproductive rights [1]. There has been a growing body of evidence pointing to the prevalence of disrespect and abuse (D&A) during facility-based childbirth. D&A is a violation of women’s rights to respectful maternity care (RMC), has a deterrent effect on the utilization of facilities for childbirth, and negatively impacts maternal and newborn health [2–6].

D&A is a complex phenomenon that includes a degree of subjectivity, as women, families, healthcare workers, and community stakeholders might define it differently. Furthermore, D&A is deeply imbedded within clinical practices and health systems [7]. This complicates intervention efforts, which must target the various contextual elements and practices that precipitate D&A [8]. Bowser and Hill described seven categories of D&A during childbirth: physical abuse, non-consented care, non-dignified care, discrimination, abandonment, and detainment in facilities [9]. In 2014, Freedman et al. defined D&A as the “﻿interactions or facility conditions that local consensus deems to be humiliating or undignified, and those interactions or conditions that are experienced as or intended to be humiliating or undignified,” a definition which captures individual instances of D&A but also the systemic factors contributing to disrespectful or abusive environments [10].

In Latin America, D&A is recognized as a component of *violencia obstétrica* (obstetric violence), a concept which has gained traction throughout the region. Obstetric violence (OV) was first codified into law in Venezuela in 2007 and has subsequently been decried by activist movements, legislative advances, and scholarly discourse in other Latin American countries [11]. OV frames disrespectful and abusive practices within the context of structural inequities and criticizes the power hierarchies imbedded in medical obstetrics. Though these analyses are critical to promoting RMC, the present article will use the term D&A instead of OV to focus the scope of discussion.

Despite the robust body of evidence for the prevalence of D&A worldwide [9, 12–14], less emphasis is placed upon identifying the drivers of the phenomenon. Country-specific analyses and solutions are needed, as successful interventions will vary greatly by local culture and context. Additionally, few existing studies of D&A pay special attention to identifying—and addressing—D&A as experienced by vulnerable patient populations.

This report is part of a larger study funded by the Fulbright Commission in Colombia, “Maternal Health Care: The Indigenous Migrant Perspective in Medellín, Colombia,” aimed at addressing these gaps to identify the manifestations and drivers of D&A as experienced by internally displaced indigenous women in Colombia. Previous studies have identified D&A within Colombian women’s experiences of hospital-based childbirth (though no true measurements of prevalence have been obtained) [15–17]. However, these studies focus mainly on making this phenomenon visible. There is a lack of data identifying the concrete drivers of D&A, which may be stunting intervention efforts.

Previous studies have indicated that migrants and refugees are particularly vulnerable to disrespectful and abusive treatment during childbirth [5, 18]. Others note that indigenous women experience language and cultural barriers that prevent the provision of RMC and facilitate D&A [19]. Colombia has faced one of the world’s most severe forced migration crises due to conflict and violence. Despite the 2016 peace agreement signed between the country’s largest armed group, the Fuerzas Armadas Revolucionarias de Colombia (FARC) and the Colombian government, there were 139,000 new displacements in 2019, bringing the total number of internally displaced persons in the country to over 5.5 million [20]. Internally displaced women of indigenous descent are an especially vulnerable group in Colombia: they are more likely to be of low socioeconomic status, are at increased risk of post-displacement trauma given historical attachment to their land and face unique cultural barriers to receiving health care. Even those indigenous women who migrate to urban regions for economic or educational reasons, rather than being forcibly displaced due to conflict, face linguistic and cultural barriers [21, 22].

The present study is the first of our knowledge to identify drivers and potential interventions to eliminate D&A against an indigenous community in Colombia. The indigenous group Embera (which in the Colombian department of Antioquia includes the subgroups Chamí, Eyabida, and Dobida) represent the largest proportion of indigenous persons (nearly 44%) in Medellín, the capital of Antioquia and the setting of this project [23, 24].

Ratcliffe’s 2013 framework, based on Bronfenbrenner’s ecological systems model, illustrates interactions between individual and systems-level factors that impact D&A [25, 26]. Under this model, D&A is affected by four interrelated subsystems: the individual and community, the healthcare professional, the healthcare facility, and national systems. Each level presents a potential target for intervention but does not exist in isolation from the other subsystems. This paper uses this framework to describe the perceived drivers of D&A against Embera women during childbirth in Colombia, and potential interventions to mitigate mistreatment.

## Methods

Qualitative data in the form of semi-structured, face-to-face interviews were collected between November 2019 and March 2020. Participants included 10 indigenous Embera women who had migrated to Medellín, Colombia, either forcibly due to the armed conflict or for economic or educational reasons and had given birth in a health facility or at home, 5 community stakeholders (including indigenous leaders and experts in indigenous health), and 6 healthcare workers (physicians and nurses). See Tables 1 and 2 for a detailed description of the participants. Participants were recruited by the research team with the help of community leaders at the *Organización Indígena de Antioquia (OIA)* and scholars at the *Universidad de Antioquia Facultad Nacional de Salud Pública (FNSP)* through the snowball effect. Throughout the interview period, data were preliminarily analyzed for emergent themes and a saturation matrix was used to determine the point at which thematic saturation was reached and thus, determine the point for interview completion [27, 28]*.* To mitigate the potential for selection bias, participants were recruited only on the basis that they be indigenous women with an institutionalized childbirth experience, not based on their prior identification as having experienced D&A [29]. Interviews lasted roughly 45 min and were facilitated in Spanish by the principal investigator. Interviews with the women were also accompanied by a female Colombian indigenous psychologist, who served as a trauma-informed cultural liaison. All the women interviewed spoke conversational Spanish, but the psychologist aided in interpreting certain phrases. A semi-structured interview guide with open-ended, non-leading questions was used to mitigate confirmation bias [30].

**Table 1:** Participant characteristics (healthcare workers and community stakeholders)

Participant category	Profession	Ethnicity	Workplace setting (if applicable)
Healthcare worker	Nurse	Colombian, non-indigenous	Community health center
Healthcare worker	OB/GYN	Colombian, non-indigenous	Academic
Healthcare worker	OB/GYN	Colombian, non-indigenous	Public hospital
Healthcare worker	OB/GYN	Colombian, non-indigenous	Academic
Healthcare worker	General physician	Colombian, non-indigenous	Indigenous health insurance agency
Healthcare worker	Nurse	Colombian, non-indigenous	Indigenous health insurance agency
Community stakeholder	Professor of public health and community psychology; expert in indigenous health systems	Colombian, non-indigenous	Academic
Community stakeholder	Psychologist, specialist in post-partum care	Colombian, non-indigenous	Private practice
Community stakeholder	Professor of obstetrics and gynecology, founder of a nonprofit organization to promote RMC	Colombian, non-indigenous	Academic; public hospital
Community stakeholder	Social worker at an indigenous health insurance company	Colombian, indigenous (Nasa)	Health insurance
Community stakeholder	Nurse; expert in obstetric violence	Colombian, non-indigenous	Academic

**Table 2:** Participant characteristics (indigenous women)

Ethnicity		Age at time of interview	Time in Medellín	Reason for displacement	Profession	Number of birth experiences	Approximate year of last childbirth
Indigenous (Chamí)	43		18 years	Work	Social work	4 (1 live birth)	2007
Indigenous (Chamí)	45		3 years	Economic opportunity	Craftsmanship	2	2007
Indigenous (Chamí)	37		25 years	Economic opportunity	Social work	1	2005
Indigenous (Chamí)	40		30 years	Economic opportunity	Not specified	2 (miscarriages)	2000
Indigenous (Chamí)	33		11 years	Education	Not specified	1	2005
Indigenous (Chamí)	32		10 years	Economic opportunity	Not specified	2	2018
Indigenous (Eyabida)	37		30 years	Economic opportunity	Professor	1	2016
Indigenous (Eyabida)	48		11 years	Armed conflict	Craftsmanship	2	1995
Indigenous (Chamí)	59		24 years	Armed conflict	Not specified	1	1994
Indigenous (Eyabida)	45		20 years	Armed conflict	Teacher	2	1995

Interviews with indigenous women discussed (1) cultural practices and ancestral knowledge surrounding maternity care and childbirth, (2) experiences of D&A during childbirth, (3) births in an indigenous community versus those in a health facility (if applicable), (4) perceived drivers of D&A, (5) indigenous women’s reasons for avoiding skilled maternity care and (6) potential interventions to improve childbirth experiences in the hospital setting. Interviews with community stakeholders and healthcare workers discussed (1) the cultural and political context of maternity care in Colombia, indigenous versus biomedical health systems, and providing intercultural care, (2) healthcare workers’ experiences witnessing D&A, (3) perceived drivers of D&A against indigenous women and (4) potential interventions to mitigate mistreatment. Interviews explored participants’ experiences without imposing medical definitions of phenomena such as D&A to mitigate chronological bias. The specific manifestations of D&A experienced by the women in this study and deterrents of skilled maternal care utilization are described elsewhere.

Audio recordings were transcribed and coded thematically in Spanish using NVivo Qualitative research software [31]. A qualitative content analysis approach was taken to identify the factors contributing to D&A toward indigenous women and potential solutions [32, 33]. A hierarchically organized codebook was developed deductively based on the Ratcliffe model and inductively based on other themes that emerged. All authors reviewed the coded transcripts and discussed ambiguities or discrepancies. Codes were organized according to emergent themes and analyzed using the Ratcliffe framework. Following data analysis, a meeting was held with study participants to validate the results obtained.

## Results

The results here are presented in two sections: first, the perceived drivers (factors contributing to the incidence) of D&A and secondly, proposed solutions or areas where interventions might be applied. Illustrative quotes are found in Table 3.

**Table 3:** Perceived drivers of D&A

Theme	Illustrative quotation, translated to English
**Individual and community-level drivers**	
Normalization of D&A and lack of empowerment	You have to view [D&A] through a special lens, because most women perceive that they were well attended. She came out with a cesarean section, she did not know why the cesarean section, she has no idea, but she was well attended, but her baby is here, everything is perfect. But when one puts on this lens and begins to see all these small details, that is where one realizes that we are immersed in a system that does not allow people to see. –*OB/GYN who practices in both academic and urban community settings*
	No, indigenous women who come from their communities I would say that very few of them are aware of their rights during childbirth. Very few. Why? One, the majority are illiterate, a huge disadvantage. Two, we don’t understand Spanish well. Three, there is the propensity for their husbands to care for them, so whatever the husbands decide goes. Indigenous women don’t have an easy way to reflect upon their experiences and think critically, this would be a disadvantage. –*33-year-old indigenous woman*
Lacking antenatal care	Most indigenous women do not have prenatal check-ups for example… they are not healthy women at all, most of them are women who have some degree of malnutrition, or they are obese. They never have prenatal check-ups because the indigenous patients in Medellín are displaced, most of the indigenous women in Medellín are displaced by violence, so they do not have routine health care because in the communities it is difficult to have access to western medicine during pregnancy. –*OB/GYN who practices in an academic hospital*
	“The indigenous women come [to the hospital], and they start to scold them. ‘Ay, mama, you are already 7 months pregnant, and you aren’t taking care.’ But if it’s a woman who came to an urban area for 5 or 8 days, from where there is no access to healthcare, well how would she have had prenatal visits? But they don’t focus on that, they start to make the mother feel neglectful or negligent without understanding that context.” –*37-year-old indigenous woman*
Indigenous cultural preferences	[Indigenous women] prefer to have children with a traditional midwife… they are embarrassed to show their bodies to a male doctor. And then all the injections, for them it is torture, I can’t even think about all the cutting. And then in the community we give birth standing up, I believe that a standing birth is less painful. ––*37-year-old indigenous woman*
	What I found very uncomfortable, I don’t know if all women do, but the physical examinations every hour were annoying especially in the middle of your pain and physical anguish. This is what indigenous women find very uncomfortable, even more if it’s a male doctor or nurse, we will always be opposed to that. –*33-year-old indigenous woman*
	[Indigenous women] arrive to an institution and have to lie down in a hospital bed, but that isn’t how birth is understood in their culture… they arrive in a place that is cold, with apparatuses and monitors that are strange to them. –*Professor of nursing and expert in D&A*
**Clinician-level drivers**	
Clinician prejudice	We fear doulas or traditional midwives, we seem them as aberrations, so we do not let them enter our institutions. We think that they are not capable, that they are not prepared, and in the worst cases, I have heard people say that they are sorceresses, witches, that they have rituals and strange things that are going to harm the pregnant women and the child. ––*Professor of nursing and expert in D&A*
	Traditional medicine is not considered, in fact, it is despised! It is seen as something that has no evidence, that it does not work, that it is even risky, so it is banned. There is no proper dialogue between these two medicines. –*OB/GYN*
	The woman is seen as an “object,” a second hand “object” who is simply a caregiver of children, a creator of children. This is one of the great limitations that men see, the man doesn’t have the same power that a woman has to procreate, so she has to be controlled. –*OB/GYN and university professor*
	I believe that neither indigenous women nor non-indigenous women are free from this situation of obstetric violence. From what I’ve managed to capture from conversations with indigenous and non-indigenous women who have experienced this process, I think that situations of obstetric violence are common, and I believe it occurs not only here in Medellín and in Colombia, but in the world in general. It’s a phenomenon that is almost global. *–Expert in indigenous health systems*
Linguistic or cultural barriers to communication	In the hospitals they don't have communication, they don't have differential care. Starting with the fact that the indigenous woman doesn't understand Spanish well… an indigenous woman who goes to a hospital is not going to tell the doctor how she is feeling or what is happening to her. [At the hospital] everybody is treated the same, but they need to train [the clinicians] to look at the difference between indigenous women and western women because of their customs, because of their culture. –48-year-old indigenous woman
	I could be telling her that her labor is progressing very well because she is dilating very well, and she is effacing very well, but for them it can feel like I am telling them that their baby is in danger because they do not know what dilation is, they do not know what effacing is … and then we add to that the fact that they do not speak Spanish very well then it becomes even more worse. –*Nurse experienced with indigenous communities*
	I think that the most important need, in the indigenous communities when they arrive here in the city or in our hospitals, is the need for communication. This becomes a barrier to care, although I believe that the care given in our hospitals, or in Medellin, is humanized as much as possible, I think that certain cultural communicative elements are not taken into account so as not to go over the heads of these other cultures when the need arises. So, many times the communicative aspects are interpreted [by clinicians] as perhaps less important, so they simply focus on the task at hand or the patient’s pain or on the procedure that has to be done, but other I think that other needs are left out due to communicative issues. –*Nurse and patient navigator for an indigenous health insurance company*
	Since she does not understand what I’m going to tell her, I do not explain anything to her and I do whatever I want with her, because even if I explained, she wouldn’t understand. -–*Professor of nursing and expert in D&A*
Lack of understanding of indigenous culture	We need to work with the indigenous community, we need to get closer to them, we need to improve our open-mindedness so that we can share knowledge and admit that they also have valuable knowledge and that ours is not the only one that dominates. Sometimes, we see indigenous communities as a difficult patient population, complicated because it isn’t easy to access them. We see their beliefs as strange, different. –*OB/GYN who practices in a public urban community hospital*
	First of all, there is a great lack of knowledge. Before judging the clinicians, I want to emphasize that they have a profound lack of knowledge of the particularities required of service to the indigenous population… They are not very solid in the subject of cultural competence and that is a huge mistake that the universities must assume responsibility for.” *–Expert in indigenous health systems*
Clinician training and medical culture	No, no, [clinicians are not thinking about human beings,] they are thinking about diseases, about organs… You can do a very simple experiment, go to a medical round and ask the doctor, "doctor, what is the name of the lady in bed 1" no, it is bed 1, but he doesn’t know the name of this man or woman. “Bed, 1, ah, the one with heart failure, yes, the one with heart failure.” What is his name? How many children does he have? Is he single? Is he married? Is he gay? is he… no, he does not know! Doctors are thinking about curing diseases but not about curing the sick, they are thinking about curing organs, curing wounds, but they are not thinking about curing the person. ––*OB/GYN who practices in both academic and urban community settings*
	There are many doctors who do not want to be [violent], but the pressure of the shift, the pressure to act quickly…they do not want to be this way, but the circumstances force them to rush and act in a way that isn’t their desire but rather learned from the social code and pressures of the hospital. –*Psychologist specializing in postpartum care*
Clinician burnout and demoralization	Our healthcare system, because of the volume, because of the precarious conditions that the clinician himself has, which leads him to an exhaustive routine that becomes something mechanical, and I no longer have in front of me a human being, a person, but instead a patient, just another number, just another person to attend to. So, we get to the point, the ends are well, the delivery goes well, the mother is healthy, but we forget the means. And in these means is the violence… a bad word, a mistreatment, not allowing the woman to speak, examining her without permission. – *OB/GYN who practices in a public urban community hospital*
	The doctor arrives, burnt out because he doesn’t earn well, because he has to work long hours, because he has to leave his family and hasn’t seen his children in a long time, and he’s been stuck in traffic and the city is collapsed … and then comes the patient who has had no prenatal care, who is sick, who has no medication, who I can’t see yet because I have 3 or 4 others and I’m overworked. It’s one thing after another, right? … It’s like a chemical formula leading to a final explosion. –*OB/GYN who practices in an academic hospital*
**Facility-level drivers**	
Inadequate infrastructure, space, and human resources	Here in Colombia, we have up 10 women in a single delivery room, often separated by only a curtain, or sometimes they are not even separated and are face to face. – *Psychologist specializing in postpartum care*
	For example, I was all alone because I wanted my family to be there, I wanted to be with my partner, with my family who were waiting for my child to arrive. But they forbade it, only I could enter. I wanted my midwife to accompany me, and they told me "this is not possible; this is not possible in this hospital." –*37-year-old indigenous woman*
Lack of accountability mechanisms	[Indigenous women don’t report D&A] because of fear. First, because many do not speak Spanish and, if they speak Spanish, they are not able to express, according to this western world, they are not able to express themselves… they cannot make themselves understood. Instead of waiting, of having patience with women who do not speak Spanish [hospital staff] start to scold them, “why are you speaking like that?” Then, of course the woman is angry and scared, so does not speak, so she prefers to keep quiet, unfortunately. –*43-year-old indigenous woman*
	Of course, I have witnessed D&A. And nothing happens, absolutely nothing because the woman is in a submissive position, she is at the mercy of the doctor, the doctor exercises authority and nothing happens, beyond the woman getting upset or asking him “why did you hit me?”, but nothing happens beyond that, there is no institutional sanction, there is no institutional supervision, absolutely nothing happens. Yes, there are the complaint and grievance boxes, but most women choose not to denounce and what they want is to leave the institution as soon as possible and never come back. It is rare that there is a complaint, and if there is a complaint, the mechanism used to resolve it is always in favor of the doctor. –––*OB/GYN who practices in both academic and urban community settings*
**Regional and national systems-level drivers**	
Lack of laws or policies	If you ask me in a general overview of the ecosystem of health care provision in Medellin, I believe that institutions have neither the training nor the desire nor the accountability from the law forcing them to [provide RMC], so they do not do it. And by not doing so they are not violating any regulation or if they do violate it, it is not a regulation that is enforced. –*–Expert in indigenous health systems*
Deprioritization of respectful, intercultural maternity care	The health care system does not have the cultural competencies required to provide care to indigenous populations, and this is happening all across the country. –*–Expert in indigenous health systems*
	We don’t have a health system that respects the will and desire of women. Instead, women have to adapt to institutional requirements and to the demands of the treating physicians. –––*OB/GYN who practices in both academic and urban community settings*

### Drivers of disrespect and abuse during childbirth

#### Individual or community-level drivers

##### Normalization of D&A

Participants identified the normalization of D&A as contributing to its incidence. Women described mistreatment during childbirth as somewhat expected or routine. Healthcare workers also described the normalization of D&A (especially verbal abuse) in the medical field—one obstetrician-gynecologist (OB/GYN), for example, recounted that his proposed intervention to combat D&A was immediately rejected for not capturing a “real” phenomenon.

##### Lack of autonomy and empowerment

Women, healthcare workers, and stakeholders alike agreed that very few indigenous women are adequately empowered to identify D&A or demand RMC. Women were unaware of their rights to RMC and noted that demanding better treatment was rare among indigenous communities who are unfamiliar with health systems and facilities. Several participants initially denied having experienced D&A, but later indicated having experienced discomfort, humiliation, or shame during childbirth. Participants also said that women’s rights, especially during childbirth, are seldom discussed in indigenous communities.

Even for those women who did identify mistreatment while in a health facility, fear and pain during labor prevented them from focusing on their quality of care. Furthermore, if they did wish to speak against D&A, language barriers prevented them from doing so. Multiple women reported that there were no accountability mechanisms, such as incident reports, that they could use to denounce D&A. Healthcare workers contradicted these claims—accountability mechanisms within health facilities do exist, but indigenous women were unaware of them or unable to access them.

##### Lacking antenatal care

Healthcare workers noted that indigenous women are more likely than non-indigenous Colombian women to arrive at a hospital in labor without having received any antenatal care (ANC). Lack of ANC contributed to poor rapport between woman and clinician and led women to feel overwhelmed and confused by the hospital birth process. Furthermore, as births without ANC are higher-risk, healthcare workers often became frustrated with women for not seeking care, chastising them for not “taking care of themselves” or even blaming them for poor outcomes.

#### Clinician-level drivers

##### Clinician prejudice

Women felt that clinicians’ biases against women or indigenous communities affected the care they received. They described being “looked at differently” and shamed for their culture or beliefs. This prejudice manifested through verbal abuse (disrespectful or derogatory comments) and the exclusion of traditional medicine (for example, prohibiting a woman from undergoing a sacred ritual or bringing the placenta home after birth, or barring traditional midwives from attending births). Healthcare workers agreed that indigenous women were subject to increased verbal abuse but attributed the exclusion of indigenous culture to facility and health system constraints. Participants also noted that even non-indigenous women were subject to D&A due to gender discrimination.

##### Linguistic or cultural barriers to communication

One of the most frequently cited drivers of D&A mentioned was barriers to communication. Some indigenous women in Medellín have limited Spanish proficiency, but virtually no clinicians speak indigenous languages. Furthermore, hospitals were never equipped with interpreters. Healthcare workers are thus unable to explain the birthing process to a woman, answer her questions, or ensure that care is delivered with cultural humility. Additionally, clinicians indicated that being unable to communicate with patients led to “dehumanization” of the patient.

In addition to language, cultural differences pose other communication barriers. Most indigenous women have little experience with healthcare systems. Medical jargon is particularly disconcerting. The hospital birth process is markedly different from births in indigenous communities, such that women are often unsure how to or to whom direct their questions.

##### Lack of understanding of indigenous culture

Participants described how childbirth is a meaningful event in indigenous communities and an important vehicle to preserving culture and ancestral knowledge. Indigenous women prefer to give birth in a kneeling or squatting position, be accompanied by a traditional midwife or healer, partake in sacred rituals and baths, and be surrounded by members of their communities.

Women complained that clinicians did not understand indigenous culture and lacked the context needed to provide intercultural maternity care. Healthcare workers and community stakeholders echoed these sentiments, indicating that neither medical education nor workplace-specific training adequately equipped clinicians with the knowledge and tools needed to provide patient-centered, culturally respectful obstetric care.

##### Clinician training and medical culture

Healthcare workers described disrespectful or abusive behaviors as routinely practiced in clinical settings. They highlighted an underemphasis on RMC in both medical training and clinical practice, noting that clinicians are neither trained in the principles of RMC nor how to implement it for diverse patients. In fact, the mention of D&A is often taboo and can alienate clinicians. Several participants also discussed the hegemonic role played by clinicians during childbirth, whose word is seen as so superior that it can never be questioned, especially by the patient.

##### Clinician burnout and demoralization

Maternal health facilities in Medellín are overcrowded, understaffed, and as described by many healthcare workers, “on the brink of collapse” due in part to recent closures of several maternity wards and an increase in patient volume. These conditions lead clinicians to feel burnt out, frustrated, and demoralized. Moreover, they have little time to provide individualized, culturally respectful care to their patients.

#### Facility-level drivers

##### Inadequate infrastructure, space, and human resources

Women described maternity wards, which often contain 8–10 beds for women in labor in a single room, separated only by thin curtains, as uncomfortable and lacking privacy. Several women who denied experiencing D&A themselves felt fearful or apprehensive upon hearing insults directed towards other women. Additionally, this lack of space makes it impossible for a woman’s family or friends to attend a birth. Indigenous women are particularly impacted by these restrictions, as births in their communities are typically attended by close family and a traditional midwife.

Additionally, clinicians described insufficient infrastructure and lack of human resources, noting the logistical difficulties to providing RMC in understaffed hospitals with large patient volumes.

#### National health systems-level drivers

##### Lack of laws or policies

Colombia, unlike other Latin American countries, does not have a law against D&A. Without a clear national policy, redressing disrespectful or abusive behavior is challenging, if not impossible. Furthermore, the lack of clear policy contributes to the normalization of D&A and makes it more difficult to integrate such issues into medical education or facility standards.

##### Devaluing of RMC

In addition to RMC lacking from clinical curricula, participants described RMC as a low priority of the Colombian health system. Intercultural care is similarly devalued. For example, many health insurances do not reimburse care given by traditional healers or midwives, which has contributed to the decline of these practices. Healthcare workers also connected inadequate infrastructure to the need for a systems-level intervention building the capacity of individual facilities and clinicians to provide respectful, culturally informed care.

#### Solutions to D&A

Drivers of D&A are “trigger points” at which interventions might be applied [34]. Many study participants suggested potential solutions to D&A based upon these triggers, which are described below using the same ecological systems framework to underscore that interventions must be multifactorial to address the complex, interrelated drivers of D&A.

##### Individuals and communities

D&A must be made visible, within the medical community, indigenous culture, and society, for any intervention to be effective. Several women underscored the need for educational campaigns and rights-based discourse aimed at increasing their autonomy and empowerment to demand respectful, intercultural care. Additionally, interventions to increase ANC among indigenous women are needed, as it presents an opportunity to provide women with information that will increase readiness for childbirth, develop rapport with clinicians, and reduce risk of poor birth outcomes.

##### Clinicians-focused interventions

Participants called for a shift in medicine, beginning in medical education, to emphasize patient-centered care and understanding that RMC is complimentary, not contradictory, to evidence-based practice. Participants emphasized their desire for clinicians to listen to patients and recognize women as active participants in their births. Participants also called for healthcare workers to be trained in indigenous culture and beliefs (including working collaboratively with traditional midwives) to provide more appropriate care for this population.

##### Facility-focused interventions

Participants argued that increased intercultural care cannot be accomplished through clinician education alone. Rather, indigenous communities must be active participants in the design and execution of any intervention, especially as RMC varies with culture. Several women suggested intercultural trainings be held between hospital administrators, indigenous leaders, healthcare workers, traditional healers, and indigenous women to help bridge this gap.

Hospital infrastructure presents an opportunity for intervention against D&A. Ideally, each woman would have a private room with space for birth companions, healthcare workers would be well-compensated and not overworked, patient volume would be small enough to allow individualized care, and interpreters would always be present. Despite the financial and logistical barriers to accomplishing such an infrastructural overhaul, smaller interventions might effectively mitigate D&A. For example, healthcare workers identified the need for facility norms for intercultural births so that all agree on alternative birth positions, allowing women to take home their placentas, herbal medicines, and, space permitting, birth companions. Healthcare workers felt uneasy about some of these interventions, suggesting that studies examine the safety of these practices before integrating them into clinical practice.

Healthcare workers also called for campaigns within hospitals to spur discourse surrounding RMC, as it currently is a controversial topic many do not wish to acknowledge. Additionally, community stakeholders noted that cultural liaisons and interpreters are often denied entry to hospitals and requested increased collaboration between facilities and indigenous health advocates.

##### National health systems-level

Participants desired the inclusion of rights-based language, accountability mechanisms against D&A, increased funding for RMC (and maternity care in general), and improved integration of indigenous and biomedical health systems into national health policy. Many proposed interventions, especially at the facility level, require funding increases to facilities in general and to programs that specifically target D&A. Participants also called for the strengthening of indigenous health systems to promote the preservation of indigenous culture and ancestral knowledge.

## Discussion

The articles of the United Nations’ 1979 Convention on the Elimination of All Forms of Discrimination against Women established the rights of women to be free from discrimination, physical or psychological violence, abuse, aggression, or coercion in all fields, including sexuality and reproduction [35]. A 2011 document affirming the universal rights of childbearing women by the White Ribbon Alliance for Safe Motherhood and a 2015 statement by the World Health Organization furthered the international discourse condemning D&A by explicitly affirming maternal health rights [2, 3]. The Colombian judicial system has agreed that the protection of sexual rights and reproductive rights includes the safeguarding of access to reproductive health services [36]. Despite the growing body of evidence detailing D&A worldwide and these calls for its eradication, local policies are not often enacted, and specific and meaningful action has not been taken. Notably, D&A experienced by internally displaced indigenous women in Colombia has not been evaluated.

The Political Constitution of Colombia of 1991 defined the nation as polyethnic and multicultural, indicating the obligation of the state and the rest of society to recognize, protect, and respect the different cultures and worldviews represented in the country. The vulnerable position of indigenous women in Colombia has also been recognized by the country’s Constitutional Court [37]. However, Colombia’s general health system (*Sistema General de Seguridad Social en Salud,* or General System of Social Security in Health) fails to fully recognize indigenous communities’ cultural beliefs, ancestral knowledge, and traditional medicine practices. It has not dismantled barriers to health care access by indigenous communities, such as those related to language and geographic barriers, among others [38]. Currently, the Indigenous System of Proprietary and Intercultural Health (*Sistema Indígena de Salud Propio Intercultural*, or SISPI) operates parallel to the general health system. A greater articulation between the two is greatly needed to address the particular needs of Colombia’s indigenous population [39].

This study’s findings illuminate a potential path towards eradicating D&A against indigenous women in Antioquia, Colombia. Drivers of D&A and points for intervention are identified, drawing from the perspectives of indigenous women, healthcare workers, and local stakeholders. Under the framework developed by Ratcliffe, the qualitative data indicates that D&A is driven by interrelated phenomena that fall under four connected subsystems: the individual and community, the clinician, the health facility, and the national health system.

Participants named individual and community-level factors as central to the incidence of D&A. Normalization of D&A during childbirth and indigenous women’s lack of autonomy and empowerment were described by each category of participants. Several emphasized that, while these phenomena exist across patient populations, indigenous women are particularly unaware of their rights within healthcare delivery spaces. Participants also described how indigenous women are “silent,” during childbirth itself and in denouncing D&A. Many highlighted that women’s rights during childbirth are seldom discussed in indigenous communities. This data agrees with findings from Colombia [37, 40] and Mexico [41] highlighting the invisibilization that often occurs surrounding mistreatment during childbirth and that D&A against indigenous women is difficult to identify by women themselves and by healthcare workers. These findings also explain data from a Mexican study wherein indigenous women are less likely to report experiencing OV during childbirth [42]. Rather than being less likely to experience D&A, it might be that indigenous women are less equipped than their nonindigenous counterparts to identify it. Indigenous women called for efforts to equip their communities with information about their rights within healthcare and mechanisms to lodge complaints against D&A.

Lack of ANC is another identified driver of D&A that contributes to poor rapport between women and their clinicians. ANC is known to be dependent upon geographical access, financial resources, and cultural beliefs [43]. Indigenous women are more likely to face barriers to accessing and receiving this care and are less likely to seek it from health facilities—many opt to receive care from traditional midwives and community healers instead and may only travel to the hospital should an emergency arise. Targeted interventions to promote ANC among indigenous women and incorporate indigenous healers into the general health system might help to provide more continuous and consistent care during the pregnancy in a way that is culturally respectful and accessible.

The second and third subsystems are the interrelated clinician-level and facility-level factors. The drivers associated with these subsystems include clinician prejudice, cultural, linguistic or cultural barriers to communication, lack of understanding of indigenous culture, clinician training and medical culture, burnout and demoralization, and inadequate infrastructure, space, and human resources. Participants demanded a review of medical curricula and urged the inclusion of a multicultural perspective promoting respect for traditional medicine and ancestral knowledge. Most health facilities in Colombia prohibit practices such as the presence of traditional midwives or healers alongside physicians during the childbirth, performing sacred rituals, and allowing the mother to bring the placenta home. Welcoming these practices will allow indigenous women to experience higher quality, more compassionate care. Additionally, hospitals must ensure that patients who do not speak Spanish have access to medical interpreters, rather than relying on friends or family to provide these services.

Hospital infrastructure was criticized by all categories of participants—overfull, understaffed facilities greatly complicate the delivery of RMC. Certain elements of the institutionalized birth experience, such as the prohibition of visits by family or friends or lack of private rooms, are a product of space and resource constraints. All women are treated the same in this regard, several clinicians explained—indigenous women are not subject to poorer conditions than non-indigenous patients. However, it is important to consider that indigenous women are less familiar with the hospital environment than their peers. Experiences deemed typical by the non-indigenous Colombian woman, such as crowded rooms, solitude during the birth process, or uncomfortable exams by male clinicians, are often viewed as D&A by indigenous women. The ultimate solution to these concerns would be greater allocation of funding and human resources to maternity wards. In particular, allowing birth companions for continuous support was cited by participants as being a major potential protective factor against D&A, and has been shown to improve maternal and infant outcomes [44]. Administrators might also consider other intermediate interventions, such as equipping clinicians with the tools (including interpreters) to provide reasonable accommodations to indigenous women and outreach efforts to familiarize indigenous women with the hospital environment during their pregnancies.

The final level is that of the health system, including policies regulating maternal care. Calls for greater promotion of RMC has led to some progress in this area—in 2017, the Ministry of Health and Social Protection called for departmental and local governments to ensure the safe, dignified, and adequate care for women during childbirth [45]. However, widespread interventions have not yet been designed or implemented and many feel that RMC remains a low priority in public policy. Our findings indicate that the first step to combatting D&A is to make it a socially recognized phenomenon in Colombia—among legislators, public health officials, clinicians, indigenous communities, and women themselves. D&A is symptomatic of a fractured health system which prevents clinicians from realizing every woman’s right to a quality, respectful, and dignified childbirth [7]. It is only by strengthening the agency of all women, especially those from vulnerable communities, the re-allocation of resources, and the creation of clear accountability mechanisms that mistreatment during childbirth will be eradicated.

### Limitations

The indigenous women who participated in this study belonged only to Embera subgroups. Those who elected to participate in the study had a higher level of Spanish proficiency and a closer proximity to non-indigenous settings (such as academia or public organizations) than indigenous women who were unreachable for recruitment, indicating potential selection bias within this study. We mitigated this bias to the fullest extent possible by recruiting participants through a central indigenous organization (OIA) and selecting an adequate number of participants such that thematic saturation was reached [29]. However, the results described may not be transferable to every Embera woman in Antioquia, or to different indigenous communities in Colombia or other countries. Despite the specificity of our study population, the women interviewed demonstrated diversity in birth facility and time period. Furthermore, they indicated that their proximity to academic and public settings enhanced their abilities to identify D&A within their experiences. Additionally, the timespan between the time of childbirth and interview varied greatly among participants, with some births having occurred many years prior to this study. Though this range illustrates the lasting effects that D&A might have on women, it also introduces the potential for recall bias. We followed established guidelines to ensure content validity despite these potential biases, including interviewing clinicians and community stakeholders in addition to indigenous women, maintaining consistency in the interview facilitator and semi-structured interview guide, continuing interviews until data saturation was reached, and following standard qualitative data analysis guidelines [46]. A meeting was held with the participants after the completion of data analysis to validate the results obtained.

Further studies are needed to assess the prevalence of D&A among indigenous women in Colombia and evaluate the implementation of interventions such as those proposed here.

## Conclusions

D&A is a fundamental violation of women’s rights to respectful maternity care and threatens global progress in improving maternal health outcomes. This is one of the first studies to evaluate indigenous women’s experiences of D&A, and the first to do so in Colombia. The results presented here indicate that indigenous women, like their non-indigenous peers, experience D&A during childbirth due to interrelated risk factors. However, they are also vulnerable to mistreatment due to added cultural and linguistic barriers and prejudice—factors which must be made central in public health agendas. Broad and meaningful action is urgently needed to ensure respectful, accessible, and dignified care during childbirth. Health systems must be bolstered to provide this care from a rights-based perspective, and interventions to mitigate D&A must be interdisciplinary and locally specific, considering the needs and wants of the very women they serve.

## Supplementary Information

**Additional file 1.**

## Data Availability

The datasets generated during this study are not publicly available due to the sensitivity of the subject matter and because participants did not consent to public availability. Anonymized data sets are available from the corresponding author upon reasonable request.

## Abbreviations

ANC: Antenatal care
D&A: Disrespect and abuse
OB/GYN: Obstetrician-gynecologist
OIA: Organización Indígena de Antioquia
OV: Obstetric violence
RMC: Respectful maternity care
SISPI: Sistema Indígena de Salud Propio Intercultural
