# Supplementary material for: Multistakeholder perspectives on the mistreatment of indigenous women during childbirth in Colombia: drivers and points for intervention
Source: BMC Pregnancy Childbirth. 2022 Mar 11;22:197. doi: 10.1186/s12884-022-04495-4 (PMC8917769; doi:10.1186/s12884-022-04495-4)
Supplement: Supplementary file 1 — Additional file 1. [file 12884_2022_4495_MOESM1_ESM.docx]

Supplemental Table 1: Perceived drivers of D&A, Original Spanish Quotations

| **Theme** | **Original quotation in Spanish** |
| --- | --- |
| **Individual and community-level drivers** | |
| Normalization of D&A and lack of empowerment | Uno tiene que tener como unos lentes especiales, una sensibilidad particular para mirar todas esas cosas, porque la mayoría de las personas perciben que la atendieron bien. Salió con una cesárea no supo porque la cesárea, no tiene ni idea, pero la atendieron bien, pero su bebé esta aquí todo está perfecto. Cuando uno se pone estos lentes y empieza a ver todos estos pequeños detalles, ahí es donde uno se da cuenta que estamos inmersos en un sistema que no permite que las personas vean. |
|  | No, las que venimos o vienen de comunidad yo diría que muy poco [tienen conocimiento de sus derechos durante el parto], muy poco. ¿Por qué? Uno, la mayoría son analfabetas, una buena desventaja ¿cierto? Dos, no entendemos el español bien. Tres, ellas siempre son propensas a que el marido las cuide y, lo que ellos decidan, entonces no tienen como es facilidad, esa forma de experimentar, de investigar, de averiguar, de ir un poco más allá, esa sería la desventaja. |
| Lacking antenatal care | La mayoría de mujeres indígenas no tienen controles prenatales por ejemplo… no son mujeres sanas del todo, entonces son mujeres que la mayoría tienen algún grado de desnutrición, o de mal nutrición o son obesas. Nunca tienen controles prenatales porque las pacientes indígenas de Medellín son mujeres desplazadas, gran parte de las mujeres indígenas de Medellín son desplazadas de la violencia entonces eso hace que ellas no tengan como una rutina de salud, de atención en salud, si ya de por si en las comunidades es difícil el acceso de la medicina occidental para el cuidado del embarazo. |
|  | Entonces llegan acá y los empiezan a regañar ‘¡ay! Mamá, usted tiene ya 7 meses, con un embarazo tan largo y no estás en control.’ Pero si es una señora que vino de por allá de un casco urbano de 5 o 8 días, donde no hay acceso de salud, pues, ¿donde va a ser el control? Entonces ellos no se enfocan en eso, entonces ya empiezan a hacer sentir a la mamá como descuidada o negligente, sin entender ese contexto. |
| Indigenous cultural preferences | Ellas prefieren tener los hijos con partera… les da pena mostrar, que les vean el cuerpo un médico hombre, no una mujer, entonces por eso, que, pues les colocan inyecciones, ósea para ellas todo eso es tortura, no me imagino lo del corte. Y en cambio en la comunidad pues es un parto de pie, yo digo que un parto de pie o de otra posición no estar acostada es menos doloroso. |
|  | Si, lo que me parecía muy incómodo, y no sé si para todas, es el tacto, esa cosa a cada hora es molesto, pero uno también en medio de su angustia, de su dolor, de su de todo, pues se deja, pero no es como tan agradable. *Y eso es lo que las mujeres indígenas les parecen más incomodo y más si es un ginecólogo o un enfermero, entonces siempre vamos a estar muy indispuestas a eso. |
|  | Ellas llegan a una institución de salud donde las acuestan en una camilla y para ellas no es la forma de concebir el nacimiento… Llegan a un lugar que para ellas es frio, con un montón de aparatos y monitores que son extraños para ellas. |
| **Provider-level drivers** | |
| Provider prejudice | Tenemos una aberración o un miedo a las dulas o a la partería, entonces no las dejamos entrar a las instituciones. Bebemos de un imaginario en el que pensamos que no son capaces, que no están preparada, que, en el peor de los casos, he escuchado términos de que son hechiceras, brujas, tienen otros ritos y cosas raras que le van a hacer daño a las gestantes y al niño. |
|  | La medicina tradicional no es considerada, no es tenida en cuenta, ¡es más! es menospreciada. Es vista como cosas que no tienen evidencia, que eso no sirve, que eso no funciona, que incluso es riesgoso, entonces esta proscrito, no hay un dialogo propiamente entre esas dos medicinas. |
|  | La mujer es un objeto, entre comillas "objeto" de segunda mano, es simplemente una cuidadora de niños, fundadora de niños y ese es una de las grandes limitantes que el hombre ve, el poder que tiene la mujer para procrear no lo tiene el hombre, entonces hay que someterla. |
|  | Yo creo que ni las mujeres indígenas ni la mujeres no indígenas están libres de esa situación de violencia obstétrica de lo que yo he podido más o menos captar a partir de diálogos con mujeres que han atravesado por ese proceso no indígenas e indígenas creo que se han dado situaciones de violencia obstétrica con cierta frecuencia y yo creo que no solamente aquí en Medellín y en Colombia, en el mundo en general eso es un fenómeno casi que global, pero sí creo que hace falta mucho más, y creo que gran parte facilitar digamos o garantizar los derechos en ese sentido de las mujeres indígenas. |
| Linguistic or cultural barriers to communication | La comunicación, o sea que capaciten porque la verdad que en los hospitales no tienen como esa, no diferencian. Empezando que la mujer indígena no entiende bien el español… una mujer [indígena] que lleven a un hospital no le va a comentar al médico como se está sintiendo o que le está pasando. Se trata a todo el mundo por igual, sí, que capaciten y se mire como la diferencia entre la mujer indígena y mujer occidental, por su costumbre, por su cultura. |
|  | Yo le puedo estar diciendo que su parto está avanzando muy bien porque está dilatando muy bien, y está borrando muy bien, pero para ellas eso es puede sentirse como que les estoy diciendo que su bebé está muy mal o algo porque no sabe que es dilatación, no sabe que es borrar… y si a eso le sumamos que de pronto no hablen muy bien el español si no la lengua materna la lengua indígena pues entonces también se vuelve peor. |
|  | Yo pienso que la necesidad más importante, en las comunidades indígenas cuando llegan acá a la ciudad o a los hospitales nuestros, es la necesidad comunicativa. Eso se convierte, de alguna forma en una barrera para los cuidados si bien yo creo que los cuidados que se dan en los hospitales nuestros, o en Medellín son digamos que de lo posible son humanizados, creo que no se tienen en cuenta ciertos elementos comunicativos culturales para no pasar por encima de esas otras culturas a la hora de la necesidad. Entonces muchas veces lo comunicativo se interpretan cosas que quizás no eran realmente la necesidad, entonces simplemente se quedan en el servicio afectado o en el procedimiento que se tiene que hacer, o en el dolor con el que se llega o el procedimiento como tal, pero otras necesidades creo que muchas veces quedan por fuera por cuestiones comunicativas. |
|  | Entonces como ella no me entiende lo que le voy a decir, entonces no le explico nada y hago lo que quiera con ella, porque así le explique o no explique no me va a entender. |
| Lack of understanding of indigenous culture | Nos falta trabajar con la comunidad indígena, nos falta acercarnos mejorar como la apertura de nuestra mente para que podamos compartir saberes y podamos admitir que ellos también tienen un conocimiento valioso y no es el nuestro el único que domina o que prevalece. A veces son comunidades que para nosotros las entendemos como comunidades difíciles, complejas porque acercarse a ellos no es fácil, porque nosotros vemos las creencias de ellos como extrañas, raras, diferentes. |
|  | Hay primero que todo un gran desconocimiento, antes de enjuiciar el comportamiento de los proveedores de salud occidentales quisiera partir de la noción de que ellos tienen un profundo desconocimiento, de las particularidades que requiere el servicio a población indígena… No están muy sólidos en el tema de competencias culturales y eso es un error muy grande, digamos que las universidades debemos asumir una gran responsabilidad sobre eso. |
| Provider training and medical culture | No, no [están pensando en seres humanos], están pensando en enfermedades, en órganos… Tú puedes hacer un experimento muy simple, ir a una ronda médica y decirle al doctor, “¿Doctor, como se llama la señora de la cama 1?” No, es la cama 1, pero él no sabe el nombre de esa mujer o de ese hombre. “La cama 1, ¡ah! el de la insuficiencia cardiaca, si el de la insuficiencia cardiaca.” ¿Como se llama? ¿Cuantos hijos tiene? ¿Es soltero? ¿Es casado? ¿Es gay? ¿Es que?... no, no sabe! Los médicos están pensando en curar enfermedades, no en curar enfermos. Están pensando en curar órganos, curar heridas, pero no están pensando en curar a la persona. |
|  | Hay muchos médicos que no quieren ser así, pero la presión del turno se acaba, esperar, hágalo rápido… Ellos no quisieran ser así, pero las circunstancias los obligan a apresurar mucho y a tomar unas conductas que no son de ellos, sino que muchas veces son aprendidas del gremio, o sea para quedar en el código del gremio. |
| Provider burnout and demoralization | Nuestro sistema de atención, por el volumen, por condiciones precarias que tiene el mismo profesional de la salud que lo van llevando a un agotamiento a una rutina, a que se vuelve algo mecánico y yo ya no tengo en frente mío a un ser humano, a una persona, sino un paciente, un número más a alguien más para atender. Y nos vamos a lo puntual que todo salga bien, el parto bien, la mamá bien, pero se nos olvidan los medios y en esos medios pienso yo que creo que ocurre la violencia… una mala palabra, un maltrato puede haber inclusive cosas físicas, no permitirle a la señora que hable, examinarla sin permiso. |
|  | Llega un médico que viene re cargado porque no gana bien, porque no es bien remunerado, porque tiene que hacer tunos prolongados, porque tiene abandonar a su familia, porque lleva mucho tiempo sin ver a sus hijos, o lo que sea, se monta al carro y entonces hay taco, y entonces la ciudad está colapsada… y entonces la paciente que no tiene controles prenatales, que está enferma, que no tiene los medicamentos, que no la puedo ver ya porque tengo otras 3 o 4, y estoy sobrepasado de trabajo pues es una cosa tras otra, cierto? … Es como una formula matemática, una formula química más bien, al final explota. |
| **Facility-level drivers** | |
| Inadequate infrastructure, space, and human resources | Acá en Colombia el sistema opera en una sala de partos, es decir puede haber hasta 10 mujeres en una sola habitación, muchas veces separadas por boina o digamos que por una cortina o no están en ocasiones separadas si no que están cara a cara. |
|  | Yo, por ejemplo, no estuve acompañada porque yo quería que mi familia estuviera aquí, que estuviera preparada con mi compañero, con mi familia que esperaba que llegara este ser. Entonces te prohíben, que solo puede entrar una persona, yo quería que mi partera me acompañara y me dijeron: "esto no es posible, eso en este hospital no es posible." |
| Lack of accountability mechanisms | [No hacen denuncias] por temor, primero por el español, porque ellas si hablan puro Embera, ósea hablan idioma Embera, pues y no hablan el castellano y, si hablan el castellano no son capaces de expresar, según para este mundo occidental no son capaces de expresar… como nosotros, como estoy hablando yo el castellano, no se hacen entender dicen ellos y, en vez de esperar, de tenerles también paciencia a mujeres que no hablan español las empiezan a regañar, ¿usted porque está hablando así? Hable bien, entonces claro, la mujer con rabia, con mucho miedo no habla, entonces prefiere mejor callar y seguir, lamentablemente. |
|  | Si claro, claro lo he visto. No pasó nada, absolutamente nada porque la mujer está en una posición de sumisión está a merced del médico, el medico ejerce una autoridad y no pasa nada, mas allá de la mujer disgustarse o decirle ¿usted porque me pego?, pero no pasa más de ahí no hay una sanción institucional no hay una supervisión institucional no pasa absolutamente nada. Si, están los buzones de quejas y reclamos, pero digamos que la mayoría de mujeres opta por no denunciar y o que quieren es salir lo más rápido posible de esa institución y no volver, entonces no, ese... no se da frecuentemente que haya una denuncia, y si se da la denuncia digamos que el mecanismo que se emplea para resolverla siempre está a favor del medico. |
| **Regional and national systems-level drivers** | |
| Lack of laws or policies | Si tú me preguntas en una mirada general el ecosistema de prestación de servicios de Medellín yo si no creo que la mayoría de los prestadores de servicios tengan ni ese entrenamiento ni esa vocación ni la ley tampoco los está obligando a eso entonces no lo hacen y al no hacerlo casi que no están incumpliendo ninguna norma o si la incumplen no es una norma que tenga implicaciones. |
| Deprioritization of respectful, intercultural maternity care | Los prestadores de servicio no tienen tampoco las competencias culturales que se requieren para prestarles servicios de salud a los pueblos indígenas, y esto pasa en todo el país. |
|  | No hay un sistema de atención que respete la voluntad y el deseo de las mujeres si no que las mujeres se tienen que adaptar a las exigencias institucionales y a las exigencias de los médicos tratantes. |
